# Abscess of the Brain

**Published:** 1885-07

**Authors:** 


### ABSCESS OF THE BRAIN.

A CASE REPORTED FROM THE CLINIC OF THE ATLANTA MEDICAL COLLEGE, SERVICE OF W. F. WESTMORELAND, M. D., PROFESSOR OF SURGERY, ATLANTA, GA.

Reported for THE ATLANTA MEDICAL A D SURGICAL JOURNAL.

W. C., age 26 years, presented himself to the clinic October 13, suffering with severe pain in the head, well marked stupor and disposition to coma. He gives the following history: Four years ago, in a fight, he was struck on the head with a hoe. The blow fractured his skull and rendered him senseless at the time, and for two weeks he was unconscious. He says that after that time he began to grow better, and was soon able to attend to his duties, but that the wound made with the hoe never healed. He says there has been a constant discharge from the wound all the time, but there has been very little pain until recently. It is a well known fact, gentlemen, that abscess of the brain sometimes comes on a considerable time after injury. Horner mentions an instance where there was an interval of nearly twelve months from the infliction of the violence until the abscess appeared. Sir Everard Home mentions a case where nineteen months elapsed. There are examples to be found in Gross' Surgery where three years elapsed from the time of injury until the abscess appeared. In this case it is a little over four years since the injury was inflicted, and he has been suffering for a few days only with well marked symptoms of abscess of the brain. There is one thing that may possibly influence this trouble. The patient had syphilis and was treated for it before the reception of the injury to the head, and that may possibly influence the trouble and prevent the healing. There may be a piece of necrosed bone that has kept the discharge up. We will cut down upon the bone and then we can determine whether we have necrosis or not. We simply make the existing opening in the scalp larger, and we find an opening in the cranium that has existed from the time of injury. A probe is now introduced into the opening and carried towards the centre of the brain, following a fistulous track ; now with the probe introduced to the depth of four inches, you will notice that the pus flows freely. The treatment will consist in keeping the wound in the scalp open, and keeping the abscess well drained, and in consequence of the old syphilitic trouble, we will direct for him thirty grains of iodide potash, to be given after each meal. The potash should be given largely diluted with water.

Oct. 17.—The patient is suffering to-day very greatly as the result of a closure of the scalp wound and a want of free drainage. We re-open the wound and make it larger, and direct that the wound be kept dilated with an elm tent.

Oct. 24.—Patient doing very well ; abscess discharging freely and the patient free from pain ; treatment continued. From October 24th to November 28th the discharge kept up ; the patient was kept on same treatment.

Nov. 28.—Discharge has almost ceased; the probe can be introduced only three inches, showing a filling up of the cavity at least one inch.

Dec. 12.—The patient has been improving constantly, and the cavity in the brain is gradually filling ; it measures only two and a quarter inches to-day.

Jan. 12.—Patient says he is feeling very well. He is wonderfully improved; is gaining flesh daily. The cavity has filled so much that the probe will only pass in one and a half inches. There is still a slight discharge.

Feb. 6.—Patient says he has felt perfectly well until a day or two ago, when some slight pain began at the old sore. The wound has healed entirely. We make an opening through the scalp to relieve this pain and find a slight amount of pus tinged with blood. The probe that once could be introduced four inches into the brain will not pass in at all. We have every reason to believe that the patient will soon be well.

Feb. 20.—To-day we find the wound entirely healed, with no pain at all. Every symptom has subsided. There has not been the slightest trouble since his last appearance; we therefore dismiss the patient as cured.

